# Sexual Harassment of Cisgender Women University Students: How the Gender Composition of Colleges Relates to Differential Prevalence

**DOI:** 10.3390/ijerph22071146

**Published:** 2025-07-19

**Authors:** Natalie Q. Poole, Christopher J. Cannon, Amy L. Gabriel, Emma J. Briles, Matt J. Gray

**Affiliations:** 1 Department of Psychology, University of Wyoming, Laramie, WY 82071, USA; npoole1@uwyo.edu (N.Q.P.); agabrie2@uwyo.edu (A.L.G.); 2 Department of Psychology, Western Kentucky University, Bowling Green, KY 42101, USA

**Keywords:** sexual harassment, gender harassment, college students, prevention

## Abstract

Gender harassment is the most common variant of sexual harassment and is often seen in male-dominated workplaces. Importantly, persistent gender harassment produces pronounced impairment in psychological and occupational domains. The current study aimed to examine the degree to which male dominated academic degree programs are associated with higher rates of sexual harassment compared to female dominated programs. Four academic fields were chosen for their gender disparity—business, engineering, health sciences, and education. Consistent with hypotheses, cisgender women students in the college of engineering were most likely to experience gender harassment by peers and faculty. Implications and future directions are discussed.

## 1. Introduction

Sexual harassment is defined as behavior that is derogatory towards an individual based on their gender [1]. According to Cortina and Areguin [2], there are several conceptual variants of sexual harassment. Specifically, the Tripartite Model of Sexual Harassment describes three primary categories—*sexual coercion*, *unwanted sexual attention*, and *gender harassment* [3]. Sexual coercion refers to suggestions of contingent sexual favors in exchange for a reward (e.g., receiving a passing grade in class if sexual favor is completed) or avoidance of a threat (e.g., receiving a poor recommendation letter if sexual favor is not performed). Unwanted sexual attention describes unwelcome sexual comments, touching, and pressure to date or have sex. Unwanted sexual attention and sexual coercion at times have conceptual overlap with sexual assault (e.g., non-consensual kissing or hugging, groping). Finally, gender-related harassment concerns communication that demeans an individual based on gender. This includes obscenity and vulgarity in terms of humor or images, negative comments about the ability of an individual based on their gender, or insinuation that an individual cannot perform adequately due to stereotypes about their gender.

While sexual coercion and unwanted sexual attention are often mistaken as the sole components of sexual harassment, they are the least common forms of such behavior. These facets are often described as *come-ons*, or clear sexual or romantic harassment toward an individual [2]. Much more commonly, individuals experience gender-related harassment, which is also labeled as gender-based *put-downs* [2]. Gender-related harassment typically appears as comments (e.g., indicating that someone is unable to complete a task-e.g., “women cannot be good engineers”) or behaviors (e.g., ignoring an individual’s ideas or contributions to a project) related to a person’s gender. 

Importantly, within the context of gender harassment, it is pivotal to note the difference between sex and gender as two distinct constructs, the common definitions of which are reductive in nature. In general, “sex” relies on biological facets, [3] and, as the authors therein note, the definition of “sex”, marked as a dichotomous variable (male and female), ignores the presence of intersex or third sex individuals. Gender can best be defined as an individual’s sense of themselves, which may or may not align with a person’s assigned sex at birth [3]. For the function of analyses constructed in this paper, the term “cisgender women” will be utilized—defined as individuals who were assigned female at birth and whose sense of self aligns with this biological “category”.

### 1.1. Sexual Harassment Prevalence and Impact

There are certain groups that are at greatest risk for sexual harassment in their work and academic settings. Unsurprisingly, women in male-dominated fields experience greater sexual harassment rates than women in other workplace environments [4]. While previous studies have identified patterns of harassment in male-dominated groups and profession more broadly, few have examined differential harassment rates in academia as a function of specific majors or academic departments [5]. Academia often has a higher proportion of men in positions of power (e.g., faculty), as well as some degree of tolerance for perpetrators of sexual harassment [6]. This may lead to increased risk of sexual harassment for female students (likely understood to be cisgender women). Indeed, it is estimated that between 20 and 50 percent of female students experience sexual harassment while enrolled in university [6]. Within the academic setting, there are notable differences between *colleges* (a collection of similar academic departments providing degree programs) within a larger university. Data acquired via the Administrator Research Campus Climate Collaborative (ARC3) from the University of Texas System and Pennsylvania State University show that female students in engineering and medical schools have higher rates of sexual harassment perpetrated by faculty than female students in non-science fields [6]. However, this study did not report on sexual harassment perpetrated by students due to concerns that student perpetrators may be in different colleges. Although understandable, an environment conducive to sexual harassment may lead peers and coworkers to perpetrate in work environments [7]. Additionally, if students are experiencing differential harassment based on their college, it may be useful to understand the experience of those students regardless of the college affiliation of the student perpetrator (i.e., a student from another college telling a female student that she should drop out of engineering). Sexual harassment perpetrated by fellow students may also look different than that perpetrated by faculty members. For example, due to the lack of a clear hierarchy, fellow students do not have the same degree of power to use sexual coercion to receive sexual favors that faculty could potentially abuse. However, students do have the ability to perpetrate other types of harassment, such as crude harassment and gender harassment [8], in potentially different ways (e.g., assigning work in a group project based on gender or gender roles). The current study aims to expand the existing literature by determining if sexual harassment perpetrated by students differentially affects cisgender women students in male-dominated colleges relative to female-dominated colleges.

Sexual harassment can have substantial adverse impacts on victims in terms of their psychological well-being and career pursuits. Although seemingly “less severe”, gender harassment has been found to lead to comparable levels of distress as sexual coercion [9], as well as having significant impact on professional and psychological well-being, even in the absence of other types of sexual harassment [10]. Notably, women in STEM careers (i.e., science, technology, engineering, and mathematics) are more likely to experience negative work climates and decreased belonging following gender-related harassment [7]. For undergraduate female students, sexual harassment from faculty and students has been associated with lower levels of motivation and aspiration towards STEM-related career goals [11]. In addition to psychological distress, this harassment from faculty may make students feel unwelcome and cause them to select a new degree path to avoid the experience of sexual harassment in the future. This is often the result of gender-based put-downs, which result in individuals feeling that they will not be supported in their career pursuits due to their gender.

Due to the psychological and professional impacts of sexual harassment in higher education, a more in-depth understanding of how and by who sexual harassment is perpetrated may have tremendous implications for sexual harassment education. Evidence that STEM programs have higher rates of sexual harassment could create targets for prevention, in turn preventing gender harassment in these programs. A decrease in gender harassment may increase the probability that cisgender women students will continue to pursue their chosen field and do so with reduced psychological distress. In turn, cisgender women students would have a greater opportunity to graduate and obtain jobs in currently male-dominated fields, which could eventually reduce the prevalence of sexual harassment in the workplace because of greater gender equity and representation.

### 1.2. Current Study

The purpose of this study is to determine if cisgender women college students may be at greater risk for sexual harassment and gender harassment, in particular, based on their academic field. This investigation will examine the sexual harassment experiences of cisgender women (i.e., women whose biological sex is consistent with their gender identity) as male-dominated fields are likely to place cisgender women students at greater risk for sexual harassment. As discussed, it is well-established that women in male-dominated vocational settings experience more sexual harassment than women in other fields [12], and due to this, it is expected that cisgender women college students in male-dominated fields and departments will be at greater risk for sexual harassment than women in other colleges and departments, similar to previous campus climate surveys [6]. Building on the findings of Johnson and colleagues [6], we also examine perpetration *by students* in addition to perpetration by faculty and staff, as cisgender women students may be more likely to experience harassment from students also based on their degree program. These findings could provide an important future target for sexual harassment preventative programming on college campuses for both faculty and students.

## 2. Materials and Methods

### 2.1. Participants

All on-campus students were invited to participate in the Sexual Misconduct Campus Climate Survey conducted at a medium-size public research university in the Rocky Mountain region of the United States during the Spring semester of 2022. A total of *N* = 2223 students participated in the survey, representing approximately 20% of the university’s on-campus population. This representation is consistent with other comparable campus climate surveys [13].

The sample was made up of 59% women, 36.9% men, and 3.3% transgender and gender non-conforming individuals. The slight overrepresentation of women seen in this sample is common amongst sexual misconduct surveys and other similar investigations. Due to the current study’s focus on sexual harassment directed towards cisgender women students and the small sample of transgender and gender non-conforming individuals, data from only cisgender women students were included in the final analyses. Additionally, in order to explore differential rates of harassment by gender representation, data from students who belong to four colleges with particularly discrepant gender representation were examined. The two colleges with the greatest representation of cisgender women students and the two colleges with the greatest representation of cisgender men students were selected. At the university, the colleges of education (73.0% women) and health sciences (74.9% women) have the highest proportion of cisgender women students, whereas the colleges of business (63.2% men) and engineering (79.2% men) have the highest proportion of cisgender men students. Student gender representation within academic colleges was identified using demographic information reported by the university. The sample for this study was determined by selecting the cisgender women students from these degree programs who completed the Sexual Misconduct Campus Climate Survey (total *N* = 614; engineering *n* = 129; business *n* = 127; education *n* = 121; health sciences *n* = 237).

In order to expand on these survey data, a brief assessment of the gender discrepancies in faculty members and students within each college was conducted. Faculty listed as “Dean” and “Associate Dean” or faculty containing “professor” in their job titles listed on college directory pages were eligible for inclusion in this analysis. Specifically, faculty were primarily men in the engineering college (83% men) and business college (80% men). Faculty were primarily female in the education college (33% men) and health sciences college (47% men). These are generally representative of the student populations of the college apart from health sciences, where faculty have higher male representation relative to students. It is important to note that faculty were categorized as either man or woman based on both names and photos from college faculty directory pages, which may not fully capture those individual’s gender expression. Individuals without photos and with gender neutral names were not included in this analysis.

### 2.2. Demographics

This sample is primarily Non-Hispanic White (81.6%), followed by Hispanic White (5.3%), Latino (2.5%), Asian/Asian American (2.0%), Black/African American (0.8%), and Native American (0.8%). A small percentage of participants identified as Multiracial (6.6%), or a race not listed (0.5%). Although the sample is primarily White, it is reflective of the university population as a whole. Student year varied such that 24.4% were first-year undergraduates, 16.6% were second-year undergraduates, 18.2% were third-year undergraduates, 17.8% were fourth-year undergraduates, and 5.0% were fifth-year or more undergraduates. Graduate students (15.0%) and professional students (2.9%) also participated in this study.

### 2.3. Measures

***Administrator Researcher Campus Climate Collaborative.*** The Administrator Researcher Campus Climate Collaborative [ARC3; 8] survey utilizes behaviorally specific questions to assess for sexual assault, sexual harassment, stalking, and intimate partner violence victimization on college campuses. In addition to assessing for victimization, this survey also examines knowledge of misconduct reporting resources, familiarity with campus resources, campus safety perceptions, and demographics. The ARC3 defines sexual harassment as exposure to sexual comments or behaviors, lewd comments, and unwanted attention by students or faculty since college enrollment. In addition to demographic questions, the following measures included in the ARC3 were utilized in the current study.

***Sexual Harassment by Faculty/Staff.*** The Department of Defense Sexual Experiences Questionnaire (SEQ-DoD) is a modified form of the Sexual Experiences Questionnaire [3,14] to measure the type and frequency of sexual harassment by members of staff. This modification is used by the ARC3 Campus Climate Survey, with reported high internal consistency (α = 0.95). The SEQ-DoD is a 19-item measure using a 5-point scale (0 = *Never* to 4 = *Many Times*), with any scores above 0 indicating the participant experienced sexual harassment by faculty and staff. Scores are broken down into four subscales—Sexist Hostility/Sexist Gender Harassment, Sexual Hostility/Crude Gender Harassment, Unwanted Sexual Attention, and Sexual Coercion. Crude Harassment refers to inappropriate sexual comments and jokes.

***Sexual Harassment by Students*.** A version of the Sexual Experiences Questionnaire [3,14] was modified to measure the type and frequency of sexual harassment from other students. The modification was used in the ARC3 Campus Climate Survey, with reported high internal consistency (α = 0.83). The modified SEQ uses 12 items and is measured on a 5-point scale (0 = *Never* to 4 = *Many Times*), with any scores above 0 indicating the participant experienced sexual harassment by students. Scores are broken down into four subscales—Sexist Hostility/Sexist Gender Harassment, Sexual Hostility/Crude Gender Harassment, Unwanted Sexual Attention, and Sexual Harassment via Electronic Communication. As students do not often hold academic power to exchange sexual favors for obtaining rewards or avoiding threats, Sexual Coercion is not measured. Instead, Sexual Harassment via Electronic Communication is used as a subscale. This is because students often communicate with each other via electronic communication (e-mail, social media, text message). This subscale is used to measure how much harassment occurs from other students through electronic means.

### 2.4. Procedure

A team of university researchers distributed an online survey based on the ARC3 Campus Climate Survey [8]. Data were collected during the Fall semester of 2022. The survey was available for all university students, including undergraduate, graduate, and professional students. Recruitment methods involved posters, flyers, an email from the University president, and announcements from student government. The recruitment system allowed for only one submission per student. All materials included a link to the survey and/or a response code that linked interested individuals to the survey website. At the outset of the survey, participants were presented with an informed consent page in which they were required to click in the affirmative to proceed with the remainder of the survey. Following this, they were presented with demographic information and survey measures. After completing the survey, participants were asked if they would like to be considered for a raffle by leaving their email address in a separate survey that was not traceable to the original survey. Random drawing prizes included: 5 $100, 4 $50, and 10 $10 cash prizes, Beats headphones, AirPods, premium tickets to university athletic events, and access to free outdoor gear rentals from the university, among others. The current study was approved by the [university name redacted for blind review] Institutional Review Board (#XXIRB-2022-302).

### 2.5. Data Analytic Plan

Due to the descriptive nature of the data (e.g., college membership and presence/type of sexual harassment) hypotheses were examined using cross tabulation of data and chi-square tests examining differential rates of harassment victimization as a function of college membership. Individuals were categorized as having experienced harassment if they endorsed any variant of harassment on at least one question on the ARC3. As the contingency tables are larger than 2 × 2 (i.e., 2 levels of harassment × 4 colleges), post hoc analyses were conducted using *z*-scores to determine which colleges and programs differ in terms of risk of sexual harassment for cisgender women students. Binary logistic regression analyses were conducted to determine incremental odds of experience harassment as a function of college status while holding demographic variables constant. Separate analyses were conducted for faculty/staff-perpetrated harassment and student-perpetrated harassment.

## 3. Results

### 3.1. Sexual Harassment from Faculty and Staff

**Figure 1:**
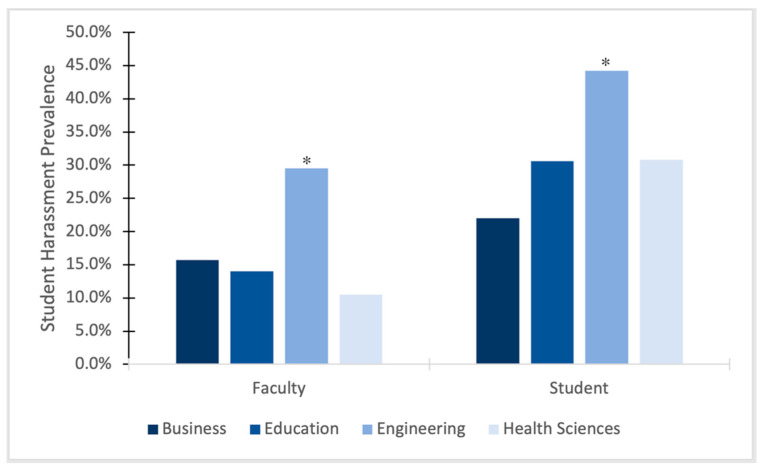
Overall sexual harassment prevalence for female students by college. * *p* < 0.05.

In comparing college women in business, engineering, education, and health sciences, data showed a significant association between college and sexual harassment from faculty overall (*X*^2^ = 22.61, *p* < 0.001; see Figure 1). Post hoc tests show that cisgender women engineering students (29.5%) experienced significantly higher rates of harassment from faculty and staff relative to students in business (15.7%), education (14.0%), and health sciences (10.5%). There were no statistically significant differences in reported harassment between or among students affiliated with these latter 3 colleges. Binary logistic regression was used to analyze the relationship between college affiliation on the probability of experiencing sexual harassment from faculty and staff. It was found that, holding demographic variables (race, age, and sexual orientation) constant, the odds of experiencing harassment if enrolled in engineering more than doubled (OR = 2.26; 90% CI [1.60, 3.19]) relative to being a student from another college.

### 3.2. Sexual Harassment from Faculty and Staff by Type of Harassment

**Figure 2:**
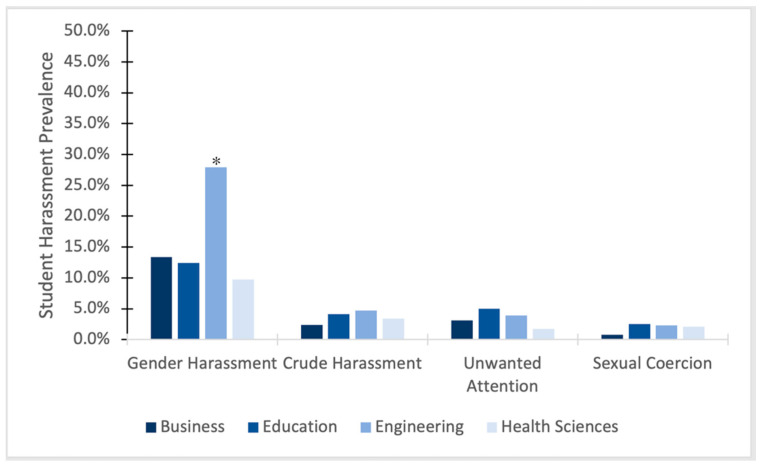
Subscales of sexual harassment perpetrated by faculty by college. * *p* < 0.05.

In terms of subtypes of harassment (see Figure 2), only one variant showed significant associations as a function of college/field. *Gender Harassment* (i.e., derogatory comments or behaviors based on one’s gender) was significantly associated with college (*X*^2^ = 23.183, *p* < 0.001). Specifically, post hoc tests showed that cisgender women students in the college of engineering (27.9%) experienced more gender harassment than cisgender women students in the college of business (13.4%), the college of education (12.4%), and the college of health sciences (9.7%). There were no significant associations between harassment and college/field on the subtypes of *Unwanted Attention* (i.e., unwanted comments or suggestions about sex or dating; *X*^2^ = 3.230; *p* = 0.357), *Coercion* (i.e., exchanging sexual favors to gain a reward or avoid a threat; *X*^2^ = 1.119; *p* = 0.753), and *Crude Harassment* (i.e., inappropriate sexual comments or jokes; *X*^2^ = 1.109; *p* = 0.775). Cisgender women engineering students experienced similarly low rates of Crude Harassment from faculty (4.7%), followed by education (4.1%), health sciences (3.4%), and then business students (2.4%). Cisgender women education students experienced similarly lower incidence of Unwanted Attention from faculty (5.0%), followed by engineering (3.9%), business (3.1%), and health sciences students (1.7%). Cisgender women education students experienced low rates of sexual coercion (2.5%), followed by engineering (2.3%), health sciences (2.1%), and business students (0.8%). Overall, prevalence of Crude Harassment, Unwanted Attention, and Sexual Coercion are quite low, which is consistent with previous literature on sexual harassment [2].

### 3.3. Sexual Harassment from Students

When comparing rates of harassment directed towards cisgender women students with college affiliation, a significant association between college and overall sexual harassment victimization from fellow students was found (*X*^2^ = 14.897, *p* = 0.002; see Figure 1). Follow-up tests revealed that cisgender women students in the engineering college (44.2%) experienced significantly higher rates of harassment from other students relative to cisgender women students in the business (22%), education (30.6%), and health sciences (30.8%) colleges. There were no statistically significant differences in reported harassment between or among students affiliated with these latter three colleges. Binary logistic regression was used to analyze the relationship between college affiliation on the probability of experiencing sexual harassment from fellow students. It was found that, holding demographic variables (race, age, and sexual orientation) constant, the odds of experiencing harassment if enrolled in engineering increased by 58% (OR = 1.58; 90% CI [1.16, 2.15]) relative to being a student from another college.

### 3.4. Sexual Harassment from Students by Type of Harassment

**Figure 3:**
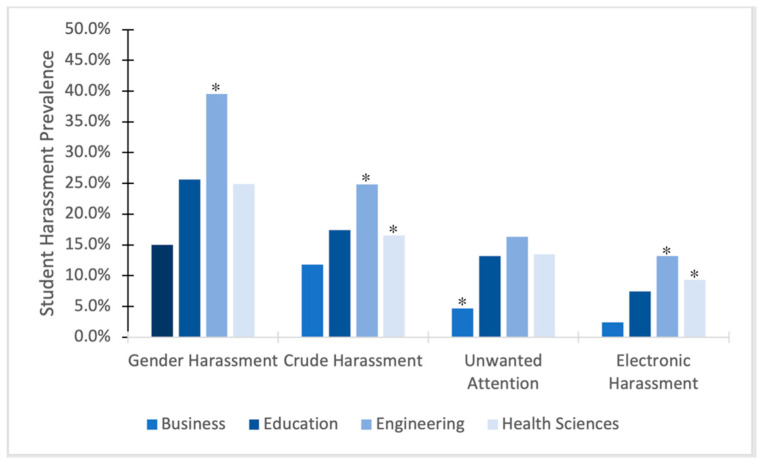
Subscales of sexual harassment perpetrated by students by college. * *p* < 0.05.

When examining subscales of student-perpetrated harassment directed at cisgender women students (see Figure 3), *Electronic Harassment* (i.e., using technology to perpetrate sexual harassment, such as making unwelcome sexual comments by text, e-mail, or social media) was significantly associated with college membership (*X*^2^ = 10.328, *p* = 0.016). Specifically, follow-up *z*-tests revealed that cisgender women students experienced significantly more electronic harassment in the engineering college (13.2%) and the health sciences college (9.3%) compared to the business college (2.4%). College of education students did not differ significantly from the other groups in this variant of harassment. *Unwanted Attention* was also significantly associated with college membership (*X*^2^ = 9.113, *p* = 0.028). Follow-up tests revealed that cisgender women students experienced significantly more Unwanted Attention in the engineering (13.2%), education (13.2%), and health sciences (13.5%) colleges compared to the college of business (4.7%). *Crude Harassment* was significantly associated with college membership (*X*^2^ = 7.821, *p* = 0.050). Post hoc tests revealed significantly higher rates of Crude Harassment directed at cisgender women students in the engineering (24.8%) and health sciences (16.5%) colleges compared to the college of business (11.8%). Lastly, *Gender Harassment* was significantly associated with college membership (*X*^2^ = 20.456, *p* < 0.001). Further examination revealed that Gender Harassment toward cisgender women students occurred at significantly higher rates in the engineering college (39.5%) compared to the college of education (25.6%), college of health sciences (24.9%) and college of business (15.0%). Gender Harassment in the college of education and health sciences was also significantly higher than Gender Harassment in the college of business.

Although most respondents did not offer optional commentary in response to open-ended questions for clarification, those who did provided observations that were concerning and consistent with empirical results. For example, one cisgender woman engineering student recognized instances of gender harassment occurring to others in the college as follows:

“Although I have personally not had to deal with sexual assault, nor have any of my friends, from my conversations with a professor, there have been a few incidents of sexism and harassment among faculty that the university did not address well…”

This indicates that some cisgender women students will be affected by gender harassment, even if they are not the target, as well as a belief that the university will not address these concerns. If students perceive a culture of tolerance to gender harassment from both the college and larger university, students may believe they will not be supported in their degree program.

Another cisgender woman engineering student mentioned that both faculty and students play a role in the culture of tolerance for gender harassment:

“The [redacted major within College of Engineering] has a serious problem with culture. Some of the faculty as well as some of the (male) students foster an environment of treating people differently because of their gender. When issues are brought up they are swept under the rug…”

This comment brings further support that cisgender women students experience different treatment from both faculty and students, and the belief that reports of gender harassment will be ignored is reiterated.

## 4. Discussion

Women working in male-dominated industries have historically been at an increased risk for sexual harassment [15]. Results from the current study confirm that this risk not only exists in male-dominated employment settings but also in male-dominated academic departments and colleges. Specifically, findings showed that cisgender women students in the male-dominated college of engineering experienced higher rates of sexual harassment from faculty compared to cisgender women students in the college of business, college of education, and college of health sciences. This pattern was typified primarily by *gender harassment*, with cisgender women students in the college of engineering experiencing more gender harassment from faculty than their cisgender female peers in the other three colleges.

Similarly, cisgender women students in the college of engineering experienced more overall *student-perpetrated* harassment relative to their peers in the college of business, college of education, and college of health sciences. This finding has not previously been established in the literature and should serve as an important guiding consideration in future prevention programming efforts. With respect to variants of harassment, cisgender women students affiliated with the college of engineering experienced significantly more student-perpetrated gender harassment than their peers affiliated with the other three colleges. Notably, cisgender women students in the college of business reported *less* student-perpetrated electronic harassment and unwanted attention compared to students in the colleges of education, health sciences, and engineering, despite being a relatively male-dominated academic college. This finding was unexpected, and future research should evaluate the degree to which it is replicable and could be designed to explore relative risk and protective factors that may differentiate business and engineering affiliations.

Interestingly, this study found that cisgender women students in the college of business reported *less* harassment than cisgender women students in the college of engineering, despite both colleges being male dominated. In fact, cisgender women students from the college of business reported the lowest rate of harassment perpetrated by fellow students compared to the other three colleges. These findings highlight discrepant levels of harassment across two different male-dominated colleges (engineering and business), and it is unclear as to why more pervasive harassment seems to occur in the college of engineering relative to the college of business, despite the similar gender breakdown of both students and faculty. Further research may clarify this discrepancy. In their research, [16] note that workplace tolerance of harassment behavior and isolated work environments have historically been associated with increased levels of harassment broadly. Cultural tolerance for sexual harassment as well as logistical differences unique to STEM fields are hypothesized as factors beyond gender that may contribute to higher rates of harassment in the college of engineering as opposed to the college of business. Open-ended survey responses from cisgender women students within male-dominated colleges suggest an awareness of the tolerance of harassment and systematic failures to address issues of harassment within these programs. This awareness further exemplifies a need to identify and address unique patterns of harassment across academic departments. Another potential factor explaining the difference in sexual harassment rates in the college of engineering versus the college of business are logistical differences unique to STEM fields. Research has indicated areas of increased isolation (e.g., isolated lab or workshop spaces) have been identified as a driver for harassment in science-dominated fields [6]. These spaces are more likely to be seen in engineering contexts rather than business contexts.

In addition to possible mechanisms that may be driving higher rates of sexual harassment in the college of engineering, there may also be mechanisms that influence lower rates of sexual harassment in the college of business. Many individuals within the college of business are required to take courses such as human resource management or business ethics. Human resource management may address relevant topics such as workplace health risks, risk management, and legal/ethical concerns [17]. It is possible that individuals receive education about workplace sexual harassment in these classes in business curricula but not in engineering studies. Research has shown that workplace training and education about sexual harassment may be promising for reducing rates of sexual harassment [18]. As such, it may be the case that decreased rates of harassment in the male-dominated college of business may be due to class content relevant to reducing rates of such harassment. Additional research analyzing course content within male-dominated departments may shed light about potential prevention avenues.

In terms of policy, Title IX of the Education Amendments Act of 1972 prohibits sexual harassment and gender-based discrimination at all institutions of higher learning that receive federal funding in the United States. Under Title IX, a school must process all complaints of sexual misconduct, regardless of where the conduct occurred, to determine whether the incident occurred in the context of an education program or activity or had continuing effects on campus or in an off-campus education program or activity. Any employee who witnesses or receives a written or verbal report or complaint of sexual misconduct that is connected to university employment or educational programs and activities must promptly report such behavior to university administrators. Subsequent investigations can result in sanctions, termination, or expulsion if allegations are substantiated.

For this reason, universities typically require students and employees during onboarding to affirm that they have read, understood, and will be compliant with the policy and that they understand that sexual harassment and other variants of sexual misconduct will not be tolerated. Nearly without exception, universities also require mandatory sexual harassment prevention training at the time of hiring (faculty/staff) or matriculation (students). Perhaps understandably, these brief trainings tend to focus on more severe or overtly sexual subtypes of harassment (i.e., crude harassment, unwanted sexual attention, or sexual coercion) and may underemphasize more subtle manifestations of gender-based differential treatment. Our data and prior research [2] affirm that gender harassment (i.e., gender-based put-downs or disrespect related to gender) is much more common and can certainly account for the continued underrepresentation of women in STEM fields. Based on the findings presented here, it is recommended that universities provide more detailed and explicit training about the types of harassment that are much more common as well as the individual and profession-wide impacts of such experiences. Importantly, training that only occurs at onboarding moments is unlikely to be effective based on the prevalence rates documented here. Increasing the frequency of harassment prevention trainings for both faculty and students as a matter of institutional policy is critically important.

Although the present study presents several strengths, it is important to recognize several potential limitations. Primarily of concern is the limited demographic width of the sample. Research indicates that women of color are more likely to experience sexual harassment because they are part of two minoritized groups [1]. Though the sample was racially representative of the university data was collected from, this sample may not be generalizable to other, more diverse universities. Further research with a wider demographic is necessary and may lend more insight into how intersectionality might play a role in sexual harassment across different college departments. In addition to racial and ethnic diversity, this sample was also limited in terms of gender. Previous studies have suggested that transgender and gender non-conforming individuals are at a higher risk of sexual assault [19], and it may be that sexual harassment functions differently for this population. An additional limitation is that proportions of faculty in each college that were men or women were determined by coding names and pictures which may not be entirely accurate. Though there certainly could be erroneous classifications at the individual level, the stark gender discrepancy of faculty in most colleges studied would likely lessen the impact of isolated misclassifications with respect to primary findings. Notably, the estimated faculty gender disparity in the college of engineering mirrors fieldwide trends, and this gendered context is associated with the highest rate of harassment in the present investigation. Finally, due to the correlational nature of the data collection, the current study is not able to explicitly identify which mechanisms may be driving differential rates of harassment. Further research is necessary to clarify the validity of hypothesized mechanisms proposed in this study.

## 5. Conclusions

The current study aimed to examine the differential rates of sexual harassment experienced by cisgender women students across two male-dominated and two cisgender women-dominated academic colleges. Results reveal the highest rates of harassment, from both students and faculty, are most prevalent in the male-dominated college of engineering. Although high rates of harassment are present in the male-dominated college of engineering, this pattern does not persist for the male-dominated college of business. Mechanisms beyond student gender representation within these colleges may be driving such patterns. Factors such as department culture, course content, and training environment may be influencing these differences, although additional research is needed to confirm the role of such mechanisms. Findings suggest the need for college-specific prevention programming and additional research examining differential rates of sexual harassment across academic departments.

## Data Availability

The dataset is available by inquiry to the corresponding author.
